# (*E*)-1-(1,3-Benzodioxol-5-yl)-4,4-di­methyl­pent-1-en-3-one

**DOI:** 10.1107/S1600536812004242

**Published:** 2012-02-10

**Authors:** Hoong-Kun Fun, Ching Kheng Quah, Mohamed I. Attia, Mohammed F. El-Behairy, Omar A. Al-Deeb

**Affiliations:** a X-ray Crystallography Unit, School of Physics, Universiti Sains Malaysia, 11800 USM, Penang, Malaysia; b Department of Pharmaceutical Chemistry, College of Pharmacy, King Saud University, Riyadh 11451, Saudi Arabia; c Medicinal and Pharmaceutical Chemistry Department, Pharmaceutical and Drug Industries Research Division, National Research Centre, 12622 Dokki, Giza, Egypt

## Abstract

In the mol­ecule of the title compound, C_14_H_16_O_3_, all non-H atoms except for one methyl C atom lie on a crystallographic mirror plane. The conformation with respect to the C=C bond [1.3465 (12) Å] is *trans*. In the crystal, mol­ecules are linked *via* C—H⋯O hydrogen bonds into *C*(5) chains propagating along [100].

## Related literature

For general background to and the pharmacological activities of the title compound, see: Pessah *et al.* (2009 ▶); Jain (2005 ▶); Medina *et al.* (2005 ▶). For the preparation of the title compound, see: Aboul-Enein *et al.* (2012 ▶). For the stability of the temperature controller used for the data collection, see: Cosier & Glazer (1986 ▶). For graph-set notation, see: Bernstein *et al.* (1995 ▶). 
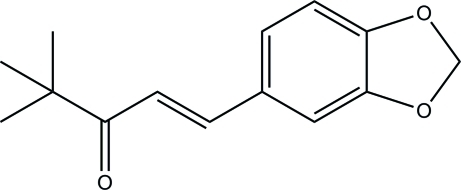


## Experimental

### 

#### Crystal data

C_14_H_16_O_3_
*M* *_r_* = 232.27
Monoclinic, 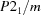

*a* = 6.5305 (1) Å
*b* = 6.6798 (1) Å
*c* = 13.7264 (2) Å
β = 96.676 (1)°
*V* = 594.72 (2) Å^3^
*Z* = 2
Mo *K*α radiation
μ = 0.09 mm^−1^
*T* = 100 K
0.42 × 0.32 × 0.25 mm

#### Data collection

Bruker SMART APEXII CCD diffractometer
Absorption correction: multi-scan (*SADABS*; Bruker, 2009 ▶) *T*_min_ = 0.963, *T*_max_ = 0.978
8645 measured reflections
2336 independent reflections
2117 reflections with *I* > 2σ(*I*)
*R*_int_ = 0.018

#### Refinement

*R*[*F* ^2^ > 2σ(*F* ^2^)] = 0.037
*wR*(*F* ^2^) = 0.110
*S* = 1.05
2336 reflections
138 parameters
All H-atom parameters refined
Δρ_max_ = 0.48 e Å^−3^
Δρ_min_ = −0.28 e Å^−3^

### 

Data collection: *APEX2* (Bruker, 2009 ▶); cell refinement: *SAINT* (Bruker, 2009 ▶); data reduction: *SAINT*; program(s) used to solve structure: *SHELXTL* (Sheldrick, 2008 ▶); program(s) used to refine structure: *SHELXTL*; molecular graphics: *SHELXTL*; software used to prepare material for publication: *SHELXTL* and *PLATON* (Spek, 2009 ▶).

## Supplementary Material

Crystal structure: contains datablock(s) global, I. DOI: 10.1107/S1600536812004242/hb6621sup1.cif

Structure factors: contains datablock(s) I. DOI: 10.1107/S1600536812004242/hb6621Isup2.hkl

Supplementary material file. DOI: 10.1107/S1600536812004242/hb6621Isup3.cml

Additional supplementary materials:  crystallographic information; 3D view; checkCIF report

## Figures and Tables

**Table 1:** Hydrogen-bond geometry (Å, °)

*D*—H⋯*A*	*D*—H	H⋯*A*	*D*⋯*A*	*D*—H⋯*A*
C2—H2*A*⋯O2^i^	0.95 (2)	2.56 (2)	3.5092 (12)	179 (1)

Symmetry code: (i) 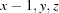.

**Fig. 1.**
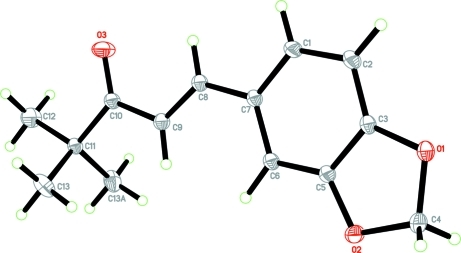
The molecular structure of the title compound showing 50% probability displacement ellipsoids for non-H atoms. Suffix A = symmetry code (x, 3/2 - y, z).

**Fig. 2.**
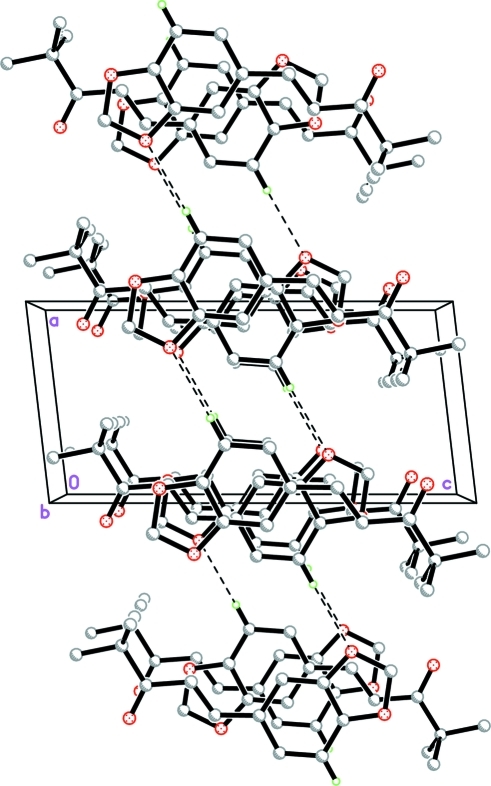
The crystal structure of the title compound, viewed along the b axis. H atoms not involved in hydrogen bonds (dashed lines) have been omitted for clarity.

## supplementary crystallographic information

### Comment

Epilepsy is a group of serious disorders of the brain characterized by excessive temporary neuronal discharge resulting in recurrent unprovoked seizures (Pessah *et al.*, 2009). It affects approximately 1% of mankind and the majority of cases are in the developing countries (Jain, 2005; Medina *et al.*, 2005). It affects about 50 million people worldwide, 1 person in 50 will develop epilepsy at some time in his life and 1 in 20 will have a single epileptic seizure. Despite the development of new methods of seizure control, chronic administration of antiepileptic drugs (AEDs) remains the treatment of choice. However, the number of non-responding patients is as high as 30% and chronic medication with currently available AEDs may result in severe side-effects and undesired drug interactions. That is why in recent years intensive research has been carried out aiming at the development of new therapeutic strategies in epilepsy. The title compound is the precursor of the recently marketed antiepileptic drug, Stiripentol. Reduction of the title compound will give Stiripentol.

The title molecule, Fig. 1, is lying on the mirror plane (symmetry code: x, -y + 3/2, z). The (1*E*)-1-(1,3-benzodioxol-5-yl)pent-1-en-3-one moiety (O1–O3/C1–C12) is exactly planar as it lies on a mirror plane. The title compound exists in *trans* configuration with respect to the C8═C9 bond [1.3465 (12) Å].

In the crystal structure, Fig. 2, molecules are linked *via* C2—H2A···O2 hydrogen bonds (Table 1) into chains along [100].

### Experimental

A 50% aqueous solution of KOH (78.5 ml) was added to a stirred solution of piperonal (11.0 g, 0.074 mol) and pinacolone (10.2 ml, 7.4 g, 0.074 mol) in methanol (200 ml). The reaction mixture was stirred and heated at 343 K for 5 h. The reaction mixture was cooled to room temperature and diluted with water (150 ml). The precipitated solid was filtered off, washed with water (50 ml) and air dried to afford the title compound which was recrystallized from ethanol as colourless blocks, m.p. = 366 K (Aboul-Enein *et al.*, 2011).

### Refinement

All H atoms were located in a difference Fourier map and refined freely with C—H = 0.952 (17)–1.026 (16) Å.

### Crystal data

**Table:**

C_14_H_16_O_3_	*F*(000) = 248
*M**_r_* = 232.27	*D*_x_ = 1.297 Mg m^−^^3^
Monoclinic, *P*2_1_/*m*	Mo *K*α radiation, λ = 0.71073 Å
Hall symbol: -P 2yb	Cell parameters from 4988 reflections
*a* = 6.5305 (1) Å	θ = 3.0–32.7°
*b* = 6.6798 (1) Å	µ = 0.09 mm^−^^1^
*c* = 13.7264 (2) Å	*T* = 100 K
β = 96.676 (1)°	Block, colourless
*V* = 594.72 (2) Å^3^	0.42 × 0.32 × 0.25 mm
*Z* = 2	

### Data collection

**Table:**

Bruker SMART APEXII CCD diffractometer	2336 independent reflections
Radiation source: fine-focus sealed tube	2117 reflections with *I* > 2σ(*I*)
Graphite monochromator	*R*_int_ = 0.018
φ and ω scans	θ_max_ = 32.7°, θ_min_ = 3.0°
Absorption correction: multi-scan (*SADABS*; Bruker, 2009)	*h* = −9→9
*T*_min_ = 0.963, *T*_max_ = 0.978	*k* = −9→10
8645 measured reflections	*l* = −20→20

### Refinement

**Table:**

Refinement on *F*^2^	Primary atom site location: structure-invariant direct methods
Least-squares matrix: full	Secondary atom site location: difference Fourier map
*R*[*F*^2^ > 2σ(*F*^2^)] = 0.037	Hydrogen site location: inferred from neighbouring sites
*wR*(*F*^2^) = 0.110	All H-atom parameters refined
*S* = 1.05	*w* = 1/[σ^2^(*F*_o_^2^) + (0.0687*P*)^2^ + 0.0862*P*] where *P* = (*F*_o_^2^ + 2*F*_c_^2^)/3
2336 reflections	(Δ/σ)_max_ = 0.001
138 parameters	Δρ_max_ = 0.48 e Å^−^^3^
0 restraints	Δρ_min_ = −0.28 e Å^−^^3^

### Special details

**Table:**

Experimental. The crystal was placed in the cold stream of an Oxford Cryosystems Cobra open-flow nitrogen cryostat (Cosier & Glazer, 1986) operating at 100.0 (1) K.
Geometry. All esds (except the esd in the dihedral angle between two l.s. planes) are estimated using the full covariance matrix. The cell esds are taken into account individually in the estimation of esds in distances, angles and torsion angles; correlations between esds in cell parameters are only used when they are defined by crystal symmetry. An approximate (isotropic) treatment of cell esds is used for estimating esds involving l.s. planes.
Refinement. Refinement of F^2^ against ALL reflections. The weighted R-factor wR and goodness of fit S are based on F^2^, conventional R-factors R are based on F, with F set to zero for negative F^2^. The threshold expression of F^2^ > 2sigma(F^2^) is used only for calculating R-factors(gt) etc. and is not relevant to the choice of reflections for refinement. R-factors based on F^2^ are statistically about twice as large as those based on F, and R-factors based on ALL data will be even larger.

### Fractional atomic coordinates and isotropic or equivalent isotropic displacement parameters (Å^2^)

**Table:**

	*x*	*y*	*z*	*U*_iso_*/*U*_eq_	
O1	−0.08309 (10)	0.7500	0.73220 (5)	0.01756 (15)	
O2	0.23387 (11)	0.7500	0.67099 (5)	0.02109 (16)	
O3	−0.10343 (12)	0.7500	0.11962 (5)	0.0284 (2)	
C1	−0.27995 (14)	0.7500	0.46990 (7)	0.01734 (18)	
C2	−0.29527 (14)	0.7500	0.57113 (7)	0.01815 (18)	
C3	−0.11215 (14)	0.7500	0.63184 (6)	0.01431 (16)	
C4	0.13580 (14)	0.7500	0.75950 (6)	0.01731 (17)	
C5	0.07776 (13)	0.7500	0.59505 (6)	0.01401 (16)	
C6	0.09473 (13)	0.7500	0.49674 (6)	0.01446 (16)	
C7	−0.08980 (13)	0.7500	0.43188 (6)	0.01338 (16)	
C8	−0.08717 (14)	0.7500	0.32577 (6)	0.01474 (16)	
C9	0.08137 (14)	0.7500	0.27781 (6)	0.01558 (17)	
C10	0.06467 (14)	0.7500	0.16901 (6)	0.01549 (17)	
C11	0.26558 (13)	0.7500	0.12174 (6)	0.01458 (16)	
C12	0.21897 (17)	0.7500	0.01018 (7)	0.0271 (2)	
C13	0.39093 (12)	0.56198 (11)	0.15410 (6)	0.02431 (16)	
H1A	−0.406 (3)	0.7500	0.4256 (12)	0.032 (4)*	
H2A	−0.424 (3)	0.7500	0.5974 (12)	0.031 (4)*	
H4A	0.1728 (15)	0.6272 (15)	0.7976 (7)	0.018 (2)*	
H6A	0.239 (2)	0.7500	0.4738 (11)	0.025 (4)*	
H8A	−0.220 (3)	0.7500	0.2883 (12)	0.034 (4)*	
H9A	0.218 (3)	0.7500	0.3117 (12)	0.032 (4)*	
H12A	0.138 (2)	0.631 (2)	−0.0117 (9)	0.041 (3)*	
H12B	0.350 (3)	0.7500	−0.0180 (12)	0.030 (4)*	
H13A	0.4241 (18)	0.5522 (18)	0.2246 (9)	0.034 (3)*	
H13B	0.3154 (19)	0.4437 (18)	0.1331 (8)	0.033 (3)*	
H13C	0.5215 (17)	0.5639 (16)	0.1247 (8)	0.026 (3)*	

### Atomic displacement parameters (Å^2^)

**Table:**

	*U*^11^	*U*^22^	*U*^33^	*U*^12^	*U*^13^	*U*^23^
O1	0.0164 (3)	0.0258 (3)	0.0112 (3)	0.000	0.0045 (2)	0.000
O2	0.0137 (3)	0.0385 (4)	0.0113 (3)	0.000	0.0022 (2)	0.000
O3	0.0141 (3)	0.0557 (6)	0.0153 (3)	0.000	0.0012 (2)	0.000
C1	0.0124 (4)	0.0258 (4)	0.0141 (4)	0.000	0.0029 (3)	0.000
C2	0.0133 (4)	0.0272 (4)	0.0149 (4)	0.000	0.0054 (3)	0.000
C3	0.0147 (4)	0.0167 (4)	0.0122 (3)	0.000	0.0046 (3)	0.000
C4	0.0173 (4)	0.0231 (4)	0.0119 (3)	0.000	0.0032 (3)	0.000
C5	0.0123 (3)	0.0173 (4)	0.0127 (3)	0.000	0.0027 (3)	0.000
C6	0.0127 (3)	0.0187 (4)	0.0126 (3)	0.000	0.0040 (3)	0.000
C7	0.0127 (3)	0.0157 (4)	0.0123 (3)	0.000	0.0034 (3)	0.000
C8	0.0142 (3)	0.0180 (4)	0.0123 (3)	0.000	0.0030 (3)	0.000
C9	0.0142 (4)	0.0210 (4)	0.0119 (3)	0.000	0.0029 (3)	0.000
C10	0.0136 (4)	0.0206 (4)	0.0127 (3)	0.000	0.0036 (3)	0.000
C11	0.0137 (3)	0.0178 (4)	0.0130 (3)	0.000	0.0045 (3)	0.000
C12	0.0222 (5)	0.0465 (7)	0.0136 (4)	0.000	0.0065 (3)	0.000
C13	0.0235 (3)	0.0215 (3)	0.0302 (3)	0.0061 (3)	0.0129 (3)	0.0056 (3)

### Geometric parameters (Å, º)

**Table:**

O1—C3	1.3685 (10)	C7—C8	1.4587 (12)
O1—C4	1.4350 (11)	C8—C9	1.3465 (12)
O2—C5	1.3706 (11)	C8—H8A	0.956 (18)
O2—C4	1.4378 (11)	C9—C10	1.4848 (12)
O3—C10	1.2214 (11)	C9—H9A	0.960 (17)
C1—C7	1.4018 (12)	C10—C11	1.5298 (12)
C1—C2	1.4046 (12)	C11—C12	1.5262 (13)
C1—H1A	0.967 (17)	C11—C13^i^	1.5363 (9)
C2—C3	1.3758 (13)	C11—C13	1.5363 (9)
C2—H2A	0.952 (17)	C12—H12A	0.985 (13)
C3—C5	1.3924 (11)	C12—H12B	0.980 (17)
C4—H4A	0.988 (10)	C13—H13A	0.969 (12)
C5—C6	1.3668 (11)	C13—H13B	0.958 (12)
C6—C7	1.4125 (12)	C13—H13C	0.985 (11)
C6—H6A	1.026 (16)		
			
C3—O1—C4	106.27 (6)	C9—C8—H8A	118.6 (10)
C5—O2—C4	106.12 (7)	C7—C8—H8A	115.0 (10)
C7—C1—C2	122.43 (8)	C8—C9—C10	121.54 (8)
C7—C1—H1A	119.6 (10)	C8—C9—H9A	122.2 (10)
C2—C1—H1A	117.9 (10)	C10—C9—H9A	116.3 (10)
C3—C2—C1	116.25 (8)	O3—C10—C9	120.97 (8)
C3—C2—H2A	120.9 (10)	O3—C10—C11	121.63 (8)
C1—C2—H2A	122.9 (10)	C9—C10—C11	117.40 (7)
O1—C3—C2	128.23 (8)	C12—C11—C10	110.16 (8)
O1—C3—C5	109.87 (8)	C12—C11—C13^i^	109.12 (5)
C2—C3—C5	121.90 (8)	C10—C11—C13^i^	109.38 (5)
O1—C4—O2	107.90 (7)	C12—C11—C13	109.12 (5)
O1—C4—H4A	108.2 (6)	C10—C11—C13	109.38 (5)
O2—C4—H4A	110.0 (6)	C13^i^—C11—C13	109.67 (8)
C6—C5—O2	127.76 (8)	C11—C12—H12A	110.1 (7)
C6—C5—C3	122.41 (8)	C11—C12—H12B	108.4 (10)
O2—C5—C3	109.83 (7)	H12A—C12—H12B	110.1 (9)
C5—C6—C7	117.46 (8)	C11—C13—H13A	113.2 (7)
C5—C6—H6A	119.0 (9)	C11—C13—H13B	110.4 (7)
C7—C6—H6A	123.5 (9)	H13A—C13—H13B	107.0 (10)
C1—C7—C6	119.55 (8)	C11—C13—H13C	109.2 (6)
C1—C7—C8	119.05 (8)	H13A—C13—H13C	107.8 (9)
C6—C7—C8	121.41 (7)	H13B—C13—H13C	109.1 (9)
C9—C8—C7	126.39 (8)		
			
C7—C1—C2—C3	0.0	C2—C1—C7—C6	0.0
C4—O1—C3—C2	180.0	C2—C1—C7—C8	180.0
C4—O1—C3—C5	0.0	C5—C6—C7—C1	0.0
C1—C2—C3—O1	180.0	C5—C6—C7—C8	180.0
C1—C2—C3—C5	0.0	C1—C7—C8—C9	180.0
C3—O1—C4—O2	0.0	C6—C7—C8—C9	0.0
C5—O2—C4—O1	0.0	C7—C8—C9—C10	180.0
C4—O2—C5—C6	180.0	C8—C9—C10—O3	0.0
C4—O2—C5—C3	0.0	C8—C9—C10—C11	180.0
O1—C3—C5—C6	180.0	O3—C10—C11—C12	0.0
C2—C3—C5—C6	0.0	C9—C10—C11—C12	180.0
O1—C3—C5—O2	0.0	O3—C10—C11—C13^i^	119.93 (5)
C2—C3—C5—O2	180.0	C9—C10—C11—C13^i^	−60.07 (5)
O2—C5—C6—C7	180.0	O3—C10—C11—C13	−119.93 (5)
C3—C5—C6—C7	0.0	C9—C10—C11—C13	60.07 (5)

Symmetry code: (i) *x*, −*y*+3/2, *z*.

### Hydrogen-bond geometry (Å, º)

**Table:**

*D*—H···*A*	*D*—H	H···*A*	*D*···*A*	*D*—H···*A*
C2—H2*A*···O2^ii^	0.95 (2)	2.56 (2)	3.5092 (12)	179 (1)

Symmetry code: (ii) *x*−1, *y*, *z*.
